# Recent advances in hydrogel-assisted treatment of malignant bone tumors

**DOI:** 10.1016/j.mtbio.2025.102088

**Published:** 2025-07-14

**Authors:** Xiangru Qian, Lian Guan, Lei Shen, Chenjun Zhai, Yedong Cheng, Guoqing Pan, Zhenhuan Jiang

**Affiliations:** a Department of Orthopedics, The Affiliated Yixing Hospital of Jiangsu University, Yixing, Jiangsu, 214200, China; b Department of Orthopedics, The Huai'an 82 Hospital, Huai'an, Jiangsu, 223001, China; c Institute for Advanced Materials, School of Materials Science and Engineering, Jiangsu University, Zhenjiang, Jiangsu, 212013, China

## Abstract

Malignant bone tumors represent a significant risk to human health, manifesting in various forms such as persistent pain, bone defects, joint dysfunction, and other complications. These factors greatly influence the prognosis and life quality of patients. Traditional tumor therapies including surgery, chemotherapy, and radiotherapy, have demonstrated effectiveness in eliminating tumor cells. However, the recurrence of tumors and bone defects caused by tumors continue to present challenges for clinicians. Therefore, there is an urgent need for innovative approaches to address these difficulties. Hydrogels are a class of promising materials with properties close to the extracellular matrix (ECM) and potential for diverse biofunctionalization. With the constant study and development of oncology and regeneration medicine, hydrogels and their composites are developing as novel implants for specialized antitumor and osteogenic treatment. This review aims to present the latest advances in the fabrication of advanced hydrogels with diverse characteristics, as well as their biological mechanisms for the treatment of malignant bone tumors, which includes both the eradication of tumor cells and the promotion of bone regeneration. Additionally, by reviewing the advancements of hydrogel-assisted treatment along with their shortcomings, a perspective will be provided.

## Graphical abstract

Biomedical applications of hydrogel biomaterials for treatment of malignant bone tumors.

**Figure:**
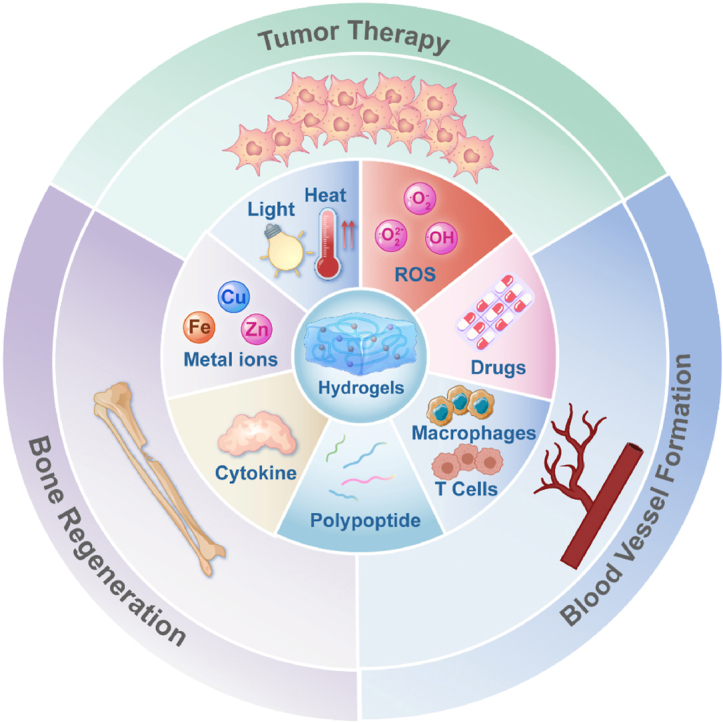

## 1 Introduction

Malignant bone tumors are bone diseases including osteosarcoma (OS), Ewing's sarcoma, and chondrosarcoma, in which OS represents the most prevalent variant [1]. OS and Ewing's sarcoma are more likely to afflict children and adolescents, and chondrosarcoma is more frequently observed in adults [[2], [3], [4]]. All these tumors tend to invade human limbs and pelvis. They can exert great distress upon patients, with initial symptoms typically manifesting as localized pain and swelling. Occasionally, pathological fractures may occur and further exacerbate the condition of patients [5]. The pathogenic mechanisms of different malignant bone tumors are still in dispute, but distal metastasis generally indicates an unfavorable prognosis in all phenotypes [6,7]. Accurate diagnosis of malignant bone tumors in the early stage still remains to be a challenge because of the inconspicuous symptoms and signs. Therefore, there has been an increased focus on enhancing treatment methodologies for malignant bone tumors in recent years.

To date, surgical excision, chemotherapy, and radiotherapy are the most common methods employed in treating malignant bone tumors [8,9]. Surgery has served as a fundamental remedy for most bone tumors in the last century, but the recurrence resulting from residual tumor cells adjacent to the surgical margin suggests the incorporation of additional treatment means. Chemotherapy is a crucial component of combined antitumor therapy, which plays a significant role in the eradication of residual tumor cells in order to inhibit undetectable metastasis. Numerous studies have demonstrated the effectiveness of chemo-agents such as doxorubicin (DOX), cisplatin (CIS), methotrexate (MTX), and cyclophosphamide (CTX) in both adjuvant chemotherapy and neoadjuvant chemotherapy [10,11]. The incorporation of chemotherapy has been shown to ameliorate the 5-year survival rate of patients with bone tumors to around 70 % compared to simple surgery alone [3]. However, the development of resistance to chemo-agents and the side effects such as nausea, fatigue, hepatotoxicity, and nephrotoxicity, imposed severe restrictions on the utilization of these drugs [12,13]. With the exception of Ewing's sarcoma, only a few malignant bone tumors respond well to radiotherapy [14], resulting in a 5-year survival rate of 60 % when patients undergo combined surgery and radiation therapy [15]. Current therapies for malignant bone tumors primarily concentrate on addressing the tumor itself and its subsequent metastasis. Consequently, the process of bone regeneration necessitates additional medical interventions.

Bone destruction typically penetrates the entire growth process of most malignant bone tumors. This prolonged destruction will induce bone defects and pathological fractures, which result in the dysfunction of adjacent limbs and joints, thereby complicating the efforts of treatment. The bone defects origin from malignant bone tumors are typically large and irregular in shape. These defects rarely heal spontaneously, necessitating the use of bone implant materials that promote bone regeneration to effectively fill and repair the affected areas. In general, the process of bone tissue regeneration requires several critical attributes: excellent biocompatibility, certain mechanical strength, adequate biodegradability, and appropriate osteoconductive and osteoinductive properties. Autogenous and allogenous bone graft are the most preferred in clinical settings due to their close resemblance to ideal materials. Especially, autografts are widely regarded as the “gold standard” in bone tissue transplantation [16,17]. However, the source of autografts may be severely limited if the bone defect is quite large, and there is also a potential risk of post-implantation infection. Furthermore, allografts face significant obstacles such as rejection reactions, disease transmission, and the high cost of materials [18,19].

These challenges have prompted researchers to investigate the implementation of artificial implant biomaterials, a field known as bone tissue engineering (BTE) [20]. Over the years, various raw materials have been employed to develop innovative composites aimed at defect repair and tumor cell elimination. For example, Wang et al. demonstrated that hydroxyapatite nanoparticles (nano-HAPs) can suppress the growth and metastasis of OS-732 cells through downregulation of FAK/PI3K/AKT pathway [21]. Free chitosan was reported to be beneficial to bone tissue reconstruction via regulating the activities of MSCs, osteoblasts and osteoclasts, and it can even promote the apoptosis of OS cells [22]. Dang et al. designed a composite scaffold consisting of metallic nanocrystals CuFeSe_2_ and bioactive glass (BG), which was shown to display dual functions in eliminating malignant bone tumor cells and accelerating the repairment of tumor-induced bone defects [23]. Three-dimensional (3D) printing is an advanced technology utilized in BTE, allowing for the personalization and customization of implants tailored to individual patients [24]. The 3D-printed scaffolds are well-suited for fitting irregular bone defects, and when combined with bioink, they can be multifunctionally engineered for tumor ablation and bone repair. Zhu et al. developed a multifunctional nanocomposite bioink composed of amine-functionalized copper-doped mesoporous bioactive glass nanoparticles (ACuMBGNs), oxidized alginate and gelatin for 3D bioprinting, which can promote the osteogenic differentiation and angiogenesis of BMSCs [25].

Although advanced materials and scaffolds have shown remarkable therapeutic efficacy in treating malignant bone tumors, a disparity still remains between these materials and the ideal biomaterials for implantation [26]. Natural polymers, including collagen and alginate, has been reported to possess potential immunogenicity, and relatively lower mechanical properties. Synthetic polymers are highly attractive biomaterials for modification in BTE, but their acidic degradation products may contaminate the microenvironment surrounding the implanted site [27]. Metals, for example, titanium and its alloy derivatives, are important orthopedic implants with favorable mechanical strength [28]. However, their insufficient bioactivity and limited biodegradation leads to a secondary surgical procedure for implant removal, which aggravates the injury and financial burden of patients [29,30]. Bioceramics, such as HA, β-TCP, and BG, commonly serve as the basis of scaffolds for incorporation and delivering bioactive ions, but they still require additional process to form composite materials in order to overcome their inherent brittleness and low fracture toughness [31,32]. The biodegradability of 3D-printed materials is relatively limited, and the durability of 3D-printed components may be influenced by thermal residual stress during the printing process [33,34]. Therefore, the exploration of new implant materials to meet the clinical demands in treating malignant bone tumors and subsequent bone defects has become a focal area of research in BTE.

Hydrogels are novel biomaterials applied in treating malignant bone tumors primarily due to their porous structure, which is formed by a hydrophilic polymer network. This structure endows hydrogels the capacity to encapsulate agents and substances that promote antitumor activity and osteogenesis [17,35]. Hydrogels possess the remarkable ability to retain water and biochemical factors, effectively mimicking the extracellular matrix. This attribute renders them the similar nature to the local environment while maintaining non-toxicity, favorable biocompatibility, and biodegradability [36,37].

The injectable capability of hydrogels represents a distinctive advantage over other biomaterials, as it facilitates the achievement of in-situ drug release and treatment within the targeted tissue, maximizing the drug administration efficiency. Generally, injectable hydrogels cause minimal invasion while traditional bone implantation requires open surgery, thereby substantially alleviating the discomfort of patients. After injection, hydrogels can completely fill irregular defects and achieve a precise fit. The drugs and cytokines encapsulated within the hydrogel will be gradually released throughout the degradation process, filling the defect cavity in a complete and uniform manner. Another advantage of hydrogels is their ability to alter physical and chemical properties in response to external stimulus [38]. These smart hydrogels can precisely regulate the release of chemotherapy drugs in line with the microenvironment of malignant bone tumors. They facilitate long-term and continuous local delivery, ensuring a sustained high therapeutic concentration of drugs. Additionally, the exceptional swelling properties of hydrogels are distinctive, enabling them to achieve high drug release efficiency. This characteristic not only prolongs the duration of drug release but also reduces the frequency of dosing when drugs are administered in the form of microneedles [39]. This approach has the potential to enhance the efficacy of chemotherapy agents, optimize the elimination of residual tumor cells, and significantly reduce systemic toxicity and side effects simultaneously. Nevertheless, the insufficient mechanical properties of hydrogels significantly restrict their potential to function as load-bearing materials for the replacement of the missing bone matrix in defects [40]. Therefore, the integration of supplementary biomaterials with hydrogels to overcome their limitations and augment their respective strengths is an emerging area of research in the field of therapies for malignant bone tumors.

In previous decades, extensive research has been conducted on hydrogels, with a primary focus on raw materials and synthesis methods. This review will provide the latest research progress of various hydrogels applied in treating malignant bone tumors, examined from alternative perspectives. This review will concentrate on distinct physicochemical properties and diverse bioactive functions of hydrogels for the treatment of malignant bone tumors, particularly on those hydrogel materials exhibiting bifunctional and multifunctional properties. This review is anticipated to inspire the development of innovative hydrogel materials, ultimately contributing to improved therapeutic efficacies for malignant bone tumors.

## 2 Functional hydrogel biomaterials

### 2.1 Hydrogels with unique physicochemical properties

Hydrogels are porous and present a three-dimensional network structure in general, hence usually serving as a load platform [37]. Various studies have demonstrated the value of hydrogel for drug release for the purpose of healing cutaneous wounds, treating internal targeted lesion, and treating many types of cancers. As the scientific understanding of malignant bone tumors constantly developed in recent decades, research on the application of hydrogel materials in treating bone tumors has also been expended because of their relatively outstanding physicochemical properties, such as injectable ability, stimuli-responsive ability, and hydrogels with enhanced mechanical strength. The injectable hydrogels facilitate smaller incisions compared to traditional surgical procedures and enable a higher local concentration of drugs due to the in situ injection method. Stimuli-responsive hydrogels can transform their physical state in response to the outer environment changes, thereby facilitating the release of contained chemotherapy drugs to infiltrate surrounding tissues adjacent to the lesion. Moreover, hydrogels with enhanced mechanical properties that do not compromise toughness have garnered significant concern in BTE, as they address the challenge posed by the relatively lower mechanical strength of hydrogels compared to other bone filling materials or scaffolds.

#### 2.1.1 Injectable hydrogels

The injectable capacity of hydrogels represents a notable advantage over other bioactive medical materials on the methods of administration. When compressed within a syringe, injectable hydrogels will maintain their biological activity due to the polymer network structure, which imparts hydrogel with adequate elasticity and plasticity. This structural integrity enables injectable hydrogels to retain water and encapsulated drugs or bioactive molecules until they are injected to targeted lesions for localized sustained release.

To date, systemic chemotherapy occupies the majority of chemotherapy regimens. While systemic chemotherapy may induce side effects or be suspended due to poor response of patients, local delivery is supposed to be the most promising alternatives. However, the localized administration of chemotherapy agents poses a challenge in dosage regulation. A rapid release of the drug will result in an accumulation of excessive drug molecules, potentially inducing local or even systematic side effects. Conversely, an insufficient concentration of drug molecules may be rapidly cleared by the blood circulation, thereby diminishing the antitumor effects. Therefore, there is a pressing need for a continuous drug delivery system to maintain the effective dosage of agents at the site of local lesions.

**Fig. 1:**
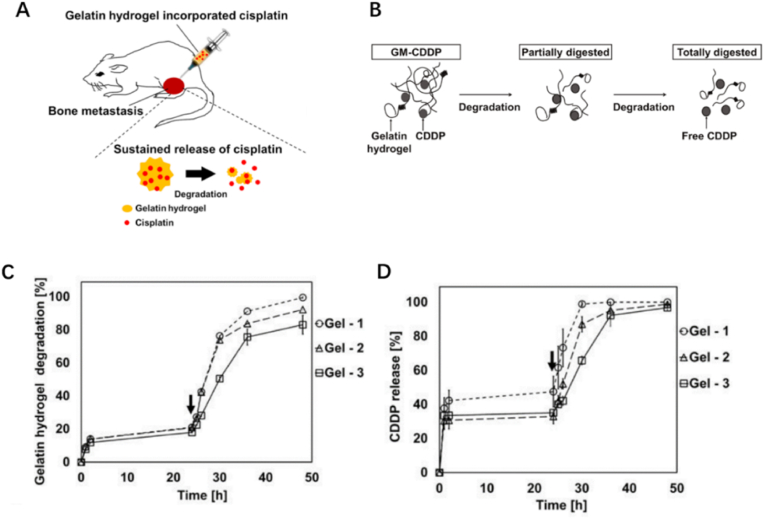
(A) Schematic illustration of the injectable GM-CDDP for antitumor effects. (B) Schematic illustration of the degradation process of GM-CDDP. (C) Time profiles of the degradation of GMs. (D) Time profiles of CDDP release form GM-CDDP. (Gel-1: dehydrothermal crosslinking at 140 °C in a vacuum oven for 24 h; Gel-2: 48 h, and Gel-3: 96 h) [41].

**Fig. 2:**
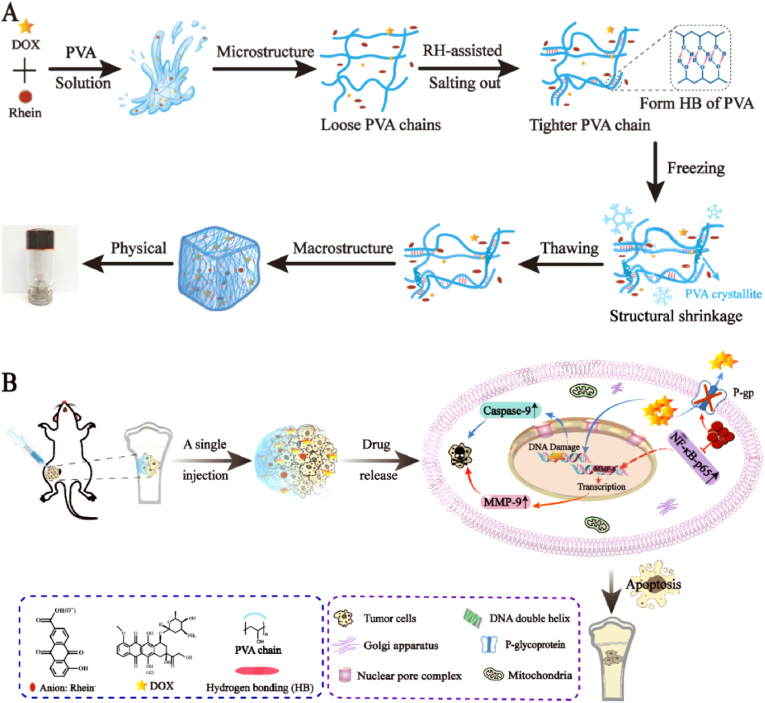
(A) The preparation process of the PVA@RH@DOX hydrogel. (B) The injectability and antitumor mechanism of PRHD [42].

Recently, Yutaro et al. reported that gelatin hydrogel microspheres incorporating cisplatin (GM-CDDP) formed via hydrothermal crosslinking was a satisfactory injectable CDDP sustained release platform, which exerted powerful antitumor effects for at least 2 weeks [41]. The hydrogel microspheres managed to maintain adequate dosage of CDDP in the tumor tissue, resulting in tumor cells apoptosis (Fig. 1A and B). What's more, organic solvents used in the production process were replaced by hydrothermal crosslinking technique in order to reduce the toxic substances. That means GM-CDDP share similar release capacity and degradability to traditional types while the safety is enhanced remarkably (Fig. 1C and D). In another study, Zhang et al. designed an injectable rhein (RH)-assisted crosslinked hydrogel PVA@RH@DOX hydrogel (PRDH), which is targeted in local DOX release for osteosarcoma [42]. PRDH is formed by mixing DOX and RH into a polyvinyl alcohol (PVA) solution, whose shear-thinning properties make the hydrogel to be injectable (Fig. 2A). RH can suppress the protein expression of matrix metalloproteinases and has no cross-resistance to DOX, significantly enhancing the antitumor effect. Moreover, the addition of RH was found to be conducive to PVA crosslinking and consequently help PRDH formation. Both K7M2 cell experiment and OS mice model confirmed that PRDH can induce OS cell apoptosis and inhibit the proliferation and metastasis of OS cells (Fig. 2B).

On the whole, injectable sustained-release hydrogels represent a significant advancement in the treatment of malignant bone tumors due to changes in administration methods. This modification substantially alleviates the suffering of patients from reiterative injections and reduces the incidence of adverse reactions associated with chemotherapeutic agents, all without compromising their antineoplastic efficacy.

#### 2.1.2 Stimuli-responsive hydrogels

Stimuli-responsive hydrogels possess the unique ability to transform their swelling performance and other natures in response to environmental stimulus such as ionic strength, pH, temperature [43,44]. These hydrogels, which can autonomously adjust their properties as if endowed with intelligence, are commonly referred to as smart hydrogels or intelligent hydrogels [45]. They hold particular promise for novel methods of drug delivery [46,47].

Due to the dynamic nature of the internal environment, precisely modulating the release of drugs or bioactive molecules within the hydrogel precisely is a tough task [48]. Once the contents in hydrogel are released uncontrollably, rapid discharge of excessive drugs may lead to local toxicity, while insufficient drug release may fail to treat targeted disease. This not only compromises the intended medical objectives but also disrupts inherent homeostasis. Stimuli-responsive hydrogels are advanced drug delivery system distinguished from other materials by its alteration behavior to adapt to dynamic microenvironment. This development offers a promising progress in therapies aimed at eradicating malignant bone tumor cells.

**Fig. 3:**
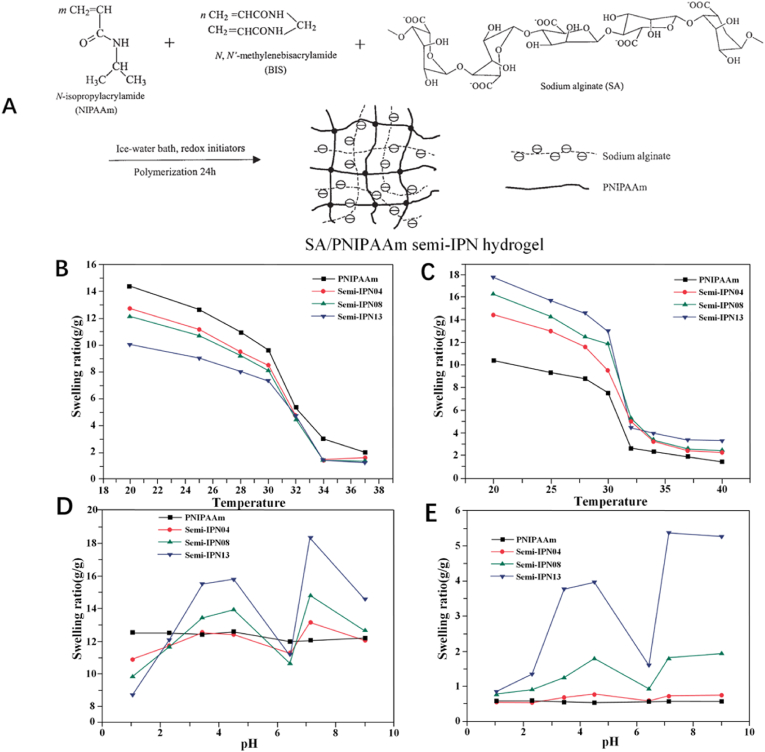
*(A) The synthesis process of SA/PNIPAAm semi-IPN hydrogel. (B-C) Swelling ratios as a function of temperature at pH 1.2 (B) and pH 7.4 (C). (D-E) Swelling ratios as a function of pH value at 25°C (D) and 37°C (E)* [49].

Zhang et al. managed to prepare a pH- and temperature-sensitive hydrogel consisting of linear sodium alginate (SA) and poly(N-isopropylacrylamide) (PNIPAAm) with the advantage of semi-interpenetrating network (semi-IPN) technique (Fig. 3A) [49]. The volume phase transition temperature (VPTT) of this hydrogel is around 33 °C. While the environmental temperature increases or decreases compared to VPTT, the SA/PNIPAAm hydrogel would swell or collapse due to the hydrophilic/hydrophobic unbalance, and the same applies to pH values (Fig. 3B–E). Malignant bone tumors require sustained chemotherapy to prevent relapse, and the chemotherapy regimen requires prompt adjustments based on the condition of patients. Innovative hydrogels such as stimuli-responsive hydrogels that can adaptively respond to fluctuating microenvironments represent a promising avenue for future research in the treatment of malignant tumors.

#### 2.1.3 High-strength hydrogels

The bone defects caused by malignant bone tumors can severely reduce the load-bearing capacity of the affected limb. Consequently, a fundamental requirement for any novel BTE materials intended for implantation in large bone defects is to possess adequate strength. Unfortunately, this characteristic often represents a significant limitation of hydrogels.

**Fig. 4:**
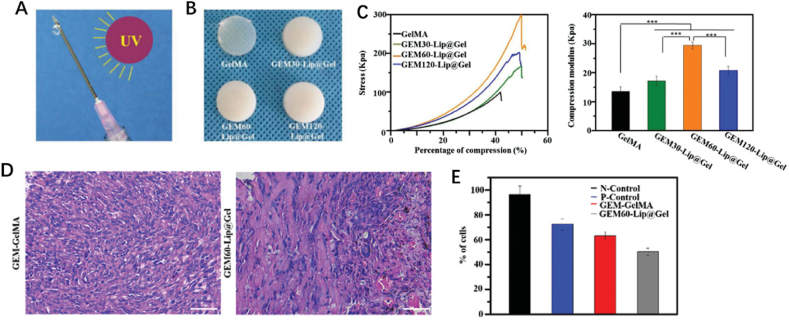
(A) The mechanism of UV-crosslinking. (B) The appearance of GelMA hydrogel and GEM-Lip@Gel with different quantities of liposome contents. (C) The compression experiments of GelMA hydrogel and GEM-Lip@Gel with different quantities of liposome contents. (D) H&E-stained images of the *in vivo* anticancer efficiency of GEM-GelMA and GEM-Lip@Gel hydrogel. (E) The percentage of MG63 cells remained [50].

On account of this dilemma, Wu et al. developed a GEM-Lip@Gel scaffold through UV-crosslinking for sustained gemcitabine (GEM) release in suppressing osteosarcoma [50]. GEM-Lip@Gel is liposome-modified, which creates a double micro-crosslinking structure because of the hydrogen bond (Fig. 4A and B). This noncovalent force can increase the level of intermolecular crosslinking of the hydrogel network. The compression modulus of GEM60-Lip@Gel was 29.49 ± 1.24 kPa, and this was more than twice than that of GelMA (Fig. 4C). This result indicated a significant improvement of the mechanical property of this hydrogel, which mainly lies in the embedding of liposome. Moreover, the hydrogel loaded with GEM was observed to exhibit antitumor efficiency both *in vitro* and *in vivo* (Fig. 4D and E).

In another study, Fu et al. successfully introduced chain entanglement into the hydrogel network composed of elastin, and greatly promoted the hardness of hydrogel to the level of cartilage without damage to its toughness [51]. This experiment provides an excellent template for subsequent development, and brings the theoretically available high-intensity hydrogel significantly closer to practical application.

### 2.2 Hydrogels with diverse bioactivities

#### 2.2.1 Hydrogels for clearing malignant bone tumors

**Fig. 5:**
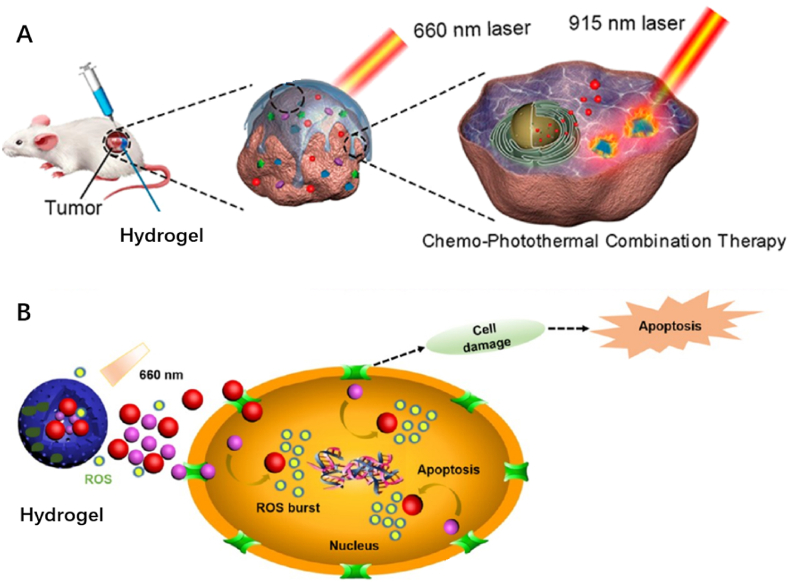
Antitumor hydrogels contribute to the death of tumor cells via PTT (A) and PDT (B).

Although the surgical excision of malignant bone tumors can remove tumor tissues and cells, the presence of rare residual cells is a great threaten to the postoperative rehabilitation, which needs further chemotherapy to obliterate them thoroughly [10]. Hydrogels are excellent drug carriers for local targeted therapy, which can reduce the side effects of chemotherapy drugs as minimal as possible via various methods. For example, enhanced chemotherapy induced by chemosensitizers and photothermal therapy (PTT) or photodynamic therapy (PDT) induced by photosensitizers are effective remedies (Fig. 5).

**Fig. 6:**
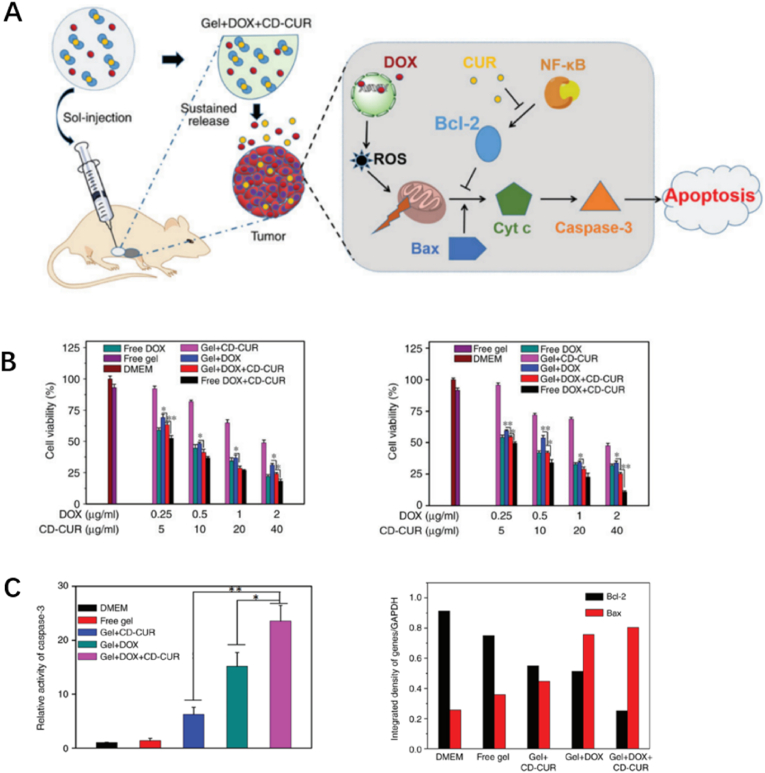
(A) The CUR and DOX were released from the sustained hydrogel into tumor sites for targeted treatment. (B) In vitro antitumor efficiencies against K-7 and Saos-2 cell of different hydrogels. (C) The antitumor efficiency reflected from protein and gene level [56].

Chemosensitizers, including resveratrol, berberine, and curcumin (CUR), are agents that enhance the sensitivity of tumor cells to chemotherapy by modulating various targets involved in mechanisms of therapy resistance [[52], [53], [54]]. In this way, the efficiency of chemotherapeutic treatments is tremendously improved by these phytochemical-based chemosensitizers and fewer side effects would be caused compared to using chemo agents alone [55]. Yang et al. selected poly(D,L-lactide-co-glycolide)-poly(ethylene-glycol)-poly(D,L-lactide-co-glycolide) (PLGA-PEG-PLGA), a thermosensitive hydrogel, as a carrier to load DOX and CD-CUR inclusion complex, which is prepared via a methanol reflux method on the basis of CUR and β-cyclodextrin (β-CD) (Fig. 6A) [56]. The inclusion complex overcomes the obstacles that natural CUR has poor solubility and instability. In vitro experiment, the dual-drug delivery hydrogel significantly potentiated the cytotoxicity and apoptotic effect of DOX (Fig. 6B). The combination of chemosensitizer and chemotherapy drug in a single hydrogel was suggested to be successful due to its most efficient antitumor effect on osteosarcoma cells compared to controlled monotherapies, which was contributed to the downregulated expression of Bcl-2 and increased level of caspase-3 (Fig. 6C). In summary, adding chemosensitizer into drug-loaded hydrogels for their conjoint sustained release is a valuable optimization aid for chemo agents and a great attempt in the treatment for malignant tumor cells.

Despite the apoptosis induced by antitumor drugs, excessive heat can also restrain the reproduction and metastasis of malignant bone tumor cells, which is the principle of PTT [57]. The energy generated from the near-infrared (NIR) light will be absorbed by photothermal conversion materials. Afterwards, transformed energy will be released out in the form of heat into tumor tissues, mediating the membrane collapse and protein denatured inside, while the normal tissue surrounding is relatively safe. PTT has achieved the treating goals that thoroughly eradicating tumor cells without excessive pain to some extent, realized by the ability of deep penetration in human tissue and minimal invasiveness. Abundant studies concentrated on NIR hydrogels against different types of tumors such as breast cancer [[58], [59], [60]], lung cancer [61], colorectal cancer have been investigated [62]. Among these investigations, scientists revealed that PTT would exhibit excellent synergistic effect with the combination of chemotherapy, greatly improving the elimination of various tumor cells, including malignant bone tumors [63,64].

**Fig. 7:**
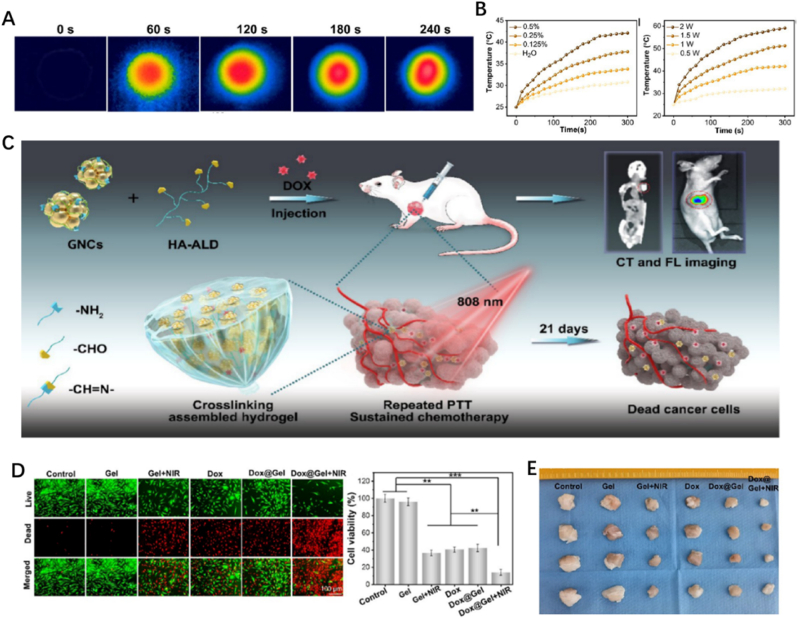
*(A) IR images of the hydrogel under 1.*0 W cm^−2^*irradiation. (B) Temperature curves of hydrogel HG-CAHs with different gradient GNC contents upon* 808 nm *laser irradiation. (C) Schematic Illustration for the hydrogel HG-CAHs treating osteosarcoma through the synergetic effect of PTT and DOX. (D) Live/dead test and survival rate of K7M2 cells. (E) Images of excised tumors* [65].

Inspired by the structure of lotus seedpods, Yang et al. invented a crosslinking-assembled hydrogel HG-CAHs, in which gold nanoclusters (GNCs) and oxidized hyaluronic acid (HA-ALD) respectively formed “lotus seeds” and “lotus stem” via dynamic amide bonds (Fig. 7C) [65]. HG-CAHs showed excellent photothermal efficiency responding to NIR irradiation because of GNCs (Fig. 7A and B), and the mechanical supportive strength has also been improved remarkably due to the lotus structure. The antitumor performance of Dox@HG-CAHs was outstanding owing to the synergetic effects produced by NIR irradiation and released DOX, along with good biocompatibility both *in vitro* and *in vivo* (Fig. 7D and E). GNCs are photothermal transducing agents while also possess strong fluorescence and computed tomography imaging effects. That means they can enhance the NIR responses and in the meantime, provide continues images of osteosarcoma treated with Dox@HG-CAHs. This added a new strategy for the diagnosis and evaluation of the prognosis, which would significantly facilitate doctors to adjust therapeutic regimen in time once applied in clinical practice.

**Fig. 8:**
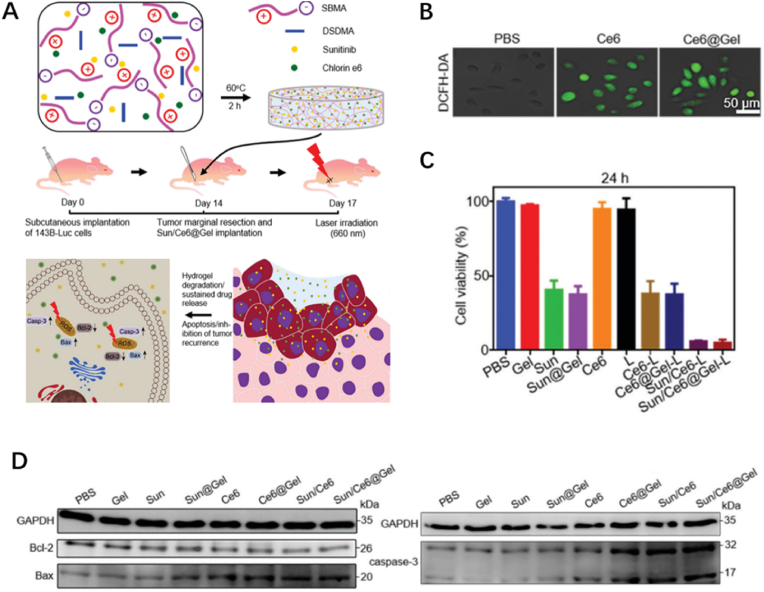
*(A) Schematic illustration of the antitumor effect of Sun and Ce6 in the Sun/Ce6@gel. (B) ROS generation (green fluorescence) of 143B cells after Ce6@Gel treatment, using DCFH-DA as the ROS probe. (C) In vitro antitumor efficiencies of Sun/Ce6@Gel on 143B cells. (D) Western blot assay showed gene and protein expression in 143B cells* [70]. (For interpretation of the references to color in this figure legend, the reader is referred to the Web version of this article.)

PDT is another light-based therapy, utilizing photosensitizers to combine with light and oxygen to generate heat and reactive oxygen species (ROS) [66]. The thermal ablation induced by heat and direct apoptosis or necrosis induced by ROS are highly effective on killing malignant tumor cells [67,68]. Besides, PDT also results in the destroy of vasculature for tumor supply and activation of antitumor immunity, giving rise to the alteration of tumor micro-environment [69]. Similar to PTT, PDT also exhibits noninvasiveness, non-toxic and spatiotemporal selectivity in medical application. Therefore, PDT has been utilized to display antitumor function singly or enhance the therapeutic effect of composite biomaterials in eradicating cancer cells. Yu et al. synthesized a redox-responsive zwitterionic hydrogel with a redox-responsive crosslinker to load sunitinib (Sun), a multi-targeted receptor tyrosine kinase (RTK) inhibitor and chlorin e6 (Ce6), a typical photosensitizer for their sustained release (Fig. 8A) [70]. Sun plays a significant role in the antitumor effect exerted by the composite hydrogel, in which this drug can inhibit tumors through suppressing RTKs, a key target involved in the tumor growth, and promoting the production of ROS generated by Ce6 (Fig. 8B). The Sun/Ce6@Gel exhibited outstanding biocompatibility and antitumor effects in experiments, which were revealed by the downregulation of Bcl-2 expression and upregulation of Bax and caspase-3 expression in osteosarcoma cells (Fig. 8C and D). The sustained release realizes the retainability of drugs, consequently inhibits the recurrence of osteosarcoma, indicating the further practice in treating malignant bone tumors after clinical excision.

Tumor immunotherapy is a highly targeted treatment method for malignant bone tumors based on the immune response from the body immune system, particularly through mechanisms of cellular immunity. A diverse array of immune cells, including T cells, dendritic cells, macrophages, and natural killer cells, not only play a significant role in the initiation and progression of tumors, but also serve as crucial targets for anti-tumor immunotherapy [71]. These immune cells infiltrate the tumor microenvironment (TME), induce apoptosis in tumor cells, and are closely associated with both tumor development and patient prognosis. Immune checkpoint inhibitors, tumor vaccines, and other immunomodulators have been reported to be promising in the treatment of malignant tumors [[72], [73], [74]]. Hydrogel is a desired localized delivery agent for tumor immunotherapy. Wang et al. developed a sequential nanocomposite hydrogel for controlled release of Nox4 inhibitor (Nox4i) and liposomal Doxorubicin (L-Dox) consists of carboxymethyl chitosan and tetrabasic polyethylene glycol, which can specifically inhibit tumor-associated fibroblasts (CAFs) secreting growth factors to orchestrate tumor progression and invasion, and effectively increase tumor T-cell infiltration to induce immunogenic cell death [75]. Additionally, tumor-infiltrating T cells (TIL-Ts) possess the ability to specifically recognize tumor antigens, thereby initiating an immune response aimed at eradicating tumor cells. However, their efficacy can be compromised by the immunosuppressive TME. He et al. invented an injectable hydrogel microsphere-integrated training court (MS-ITC) to inspire the potential of TIL-Ts for the treatment of osteosarcoma [76]. MS-ITC can sustainably release IL-7 and IL-15, providing the necessary signals for the activation and expansion of TIL-Ts. This process led to a significant increase in CD8^+^T cells and memory T cells at tumor sites, and substantially reduced the tumor volume in mice bearing osteosarcoma.

#### 2.2.2 Hydrogels for promoting bone regeneration

**Fig. 9:**
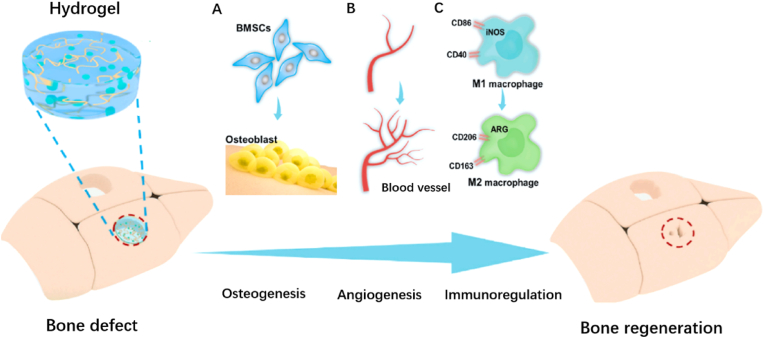
Hydrogels promote bone regeneration via osteogenesis (A), angiogenesis (B), and immunoregulation (C) [79].

Apart from the detrimental effects of the tumor itself, malignant bone tumors typically lead to significant bone defects. Despite reconstruction of the load-bearing capacity, implanted materials also play a significant role in promoting osteogenesis so as to meet the requirement of physiological function recovery. The inserted biomaterials should possess remarkable osteoinduction and osteoconduction ability aside from low immunogenicity and mechanical strength. Moreover, degradability and anti-infection ability are also taken into consideration since residual foreign materials and durable inflammation are possible to influence the prognosis of bone tissue repairment or even induce local dysfunction [77]. Hydrogel appears to be mild to human tissue because of the similarity of their structures and properties to extracellular matrix (ECM) environment [78], and have been reported to exhibit excellent capacity including antibacterial, hemostasis, anti-oxidation to accelerate the biochemical process of repairing multitudinous cases such as wounds, cartilage tissues and bone tissues. The mechanism of hydrogels facilitating bone defect repairment can be divided into two styles, the direct osteogenic styles and indirect ones. The direct methods prefer to promote the gathering and proliferation of osteoblasts and deaden the activity of osteoclasts through different cytokines. The indirect ones are more concentrated on the regulation of the osteogenic environment, particularly from the perspective of angiogenesis and immune system (Fig. 9).

Numerous studies have revealed that the process of bone tissue rebuilding, no matter which portion of the whole skeleton, demands the participation of Ca^2+^, P^5+^, BMP-2, VEGF and other cytokines or micro molecules [80,81]. For instance, stromal cell derived factor-1 (SDF-1) has a strong regulation effect on the migration of bone marrow mesenchymal stem cells (BMSCs), thus Tan et al. designed a supramolecular hydrogel consisting of Nap-Phe-Phe-Tyr-OH (NapFFY) to encapsulate SDF-1 and BMP-2 for their delivery in the periodontal bone defects [82]. This cell therapy is the same applies to post-neoplastic bone defects which require sustained release of bioactive factors. Ryoma et al. improved the production methods to photocrosslink a Gelatin methacryloyl-riboflavin hydrogel with the usage of visible wavelength light (VW) instead of UV light to protect the KUSA-A1 osteoblasts encapsulated in it [83]. Rboflavin (RF) is a photoinitiator which can release free radicals to help the radical polymerization of GelMA hydrogel, and more importantly, its absorption spectrumin exhibits resembling effects in the VW range as that in the UV range. The stiffness of hydrogel is suitable confirmed through the measurement of compressive moduli, which means the mechanical characterization is enough to fill the bone defect. Cultured in the GelMA-RF hydrogel, KUSA-A1 osteoblasts were observed to express *Ocn* mRNA remarkably, and the cells were aggregated with the formation of spheroid shape, which indicated they were highly promoted to differentiate and mature. This proved that GelMA-RF hydrogel displays excellent encapsulation and osteogenesis functions, possessing great potential in treating complex bone defects.

**Fig. 10:**
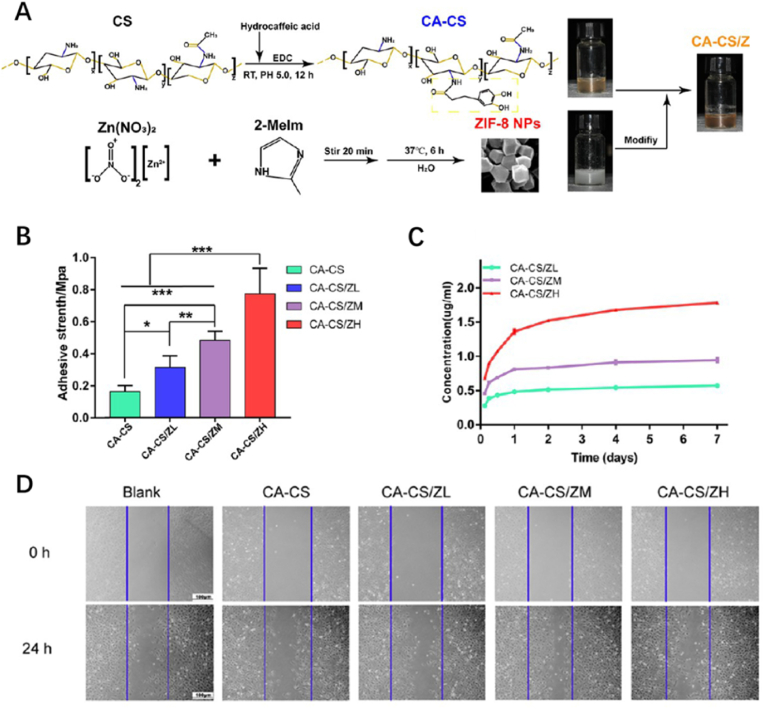
*(A) Fabrication and characterization of CA-CS/Z hydrogels. (B) The adhesive strength of different groups of hydrogels. (C) Cumulative release of Zn*^*2+*^*from the CA-CS/Z hydrogels. (D) Microscope images of HUVEVs in scratch experiments* [84].

Sufficient blood supply is a guarantee of nutrition and oxygen for bone reconstruction, especially large bone defects. Otherwise, the repairment process will be delayed or even fail. In the early stage of bone rebuilding, the growth of blood vessels is highly coupled with bone tissue formation. That means consideration should be given to both osteogenesis and vascularization before implanting biomaterials. Liu et al. reported to develop a composite hydrogel which is modified by ZIF-8 nanoparticles (NPs) and catechol functional groups on the basis of chitosan hydrogel (Fig. 10A) [84]. This catechol-functionalized chitosan nano-ZIF-8 composite hydrogel (CA-CS/Z) exhibits outstanding mechanical properties, biocompatibility, and its adhesion is much better compared to other chitosan hydrogels due to the addition of catechol groups (Fig. 10B). As a prerequisite of bone reconstruction, a suitable and stable microenvironment is formed on account of the Zn^2+^ released from ZIF-8 NPs (Fig. 10C), which not only promote the oxidation of catechol groups, generating quinones to create an alkaline environment, but also stimulate VEGF-A, one of growth factors produced by rBMSCs to secrete through paracrine effect, resulting in the enrichment of vascular endothelial cells (Fig. 10D). Moreover, the expression of *Alp*, *Runx2*, and *Col1* genes that are associated with osteogenesis was observed to be significantly up-regulated, and the secretary proteins related to osteoblasts such as ALP, OCN, and COL1 were promoted too, suggesting the distinct osteogenic reaction was quite active. CA-CS/Z hydrogel also prolonged the delivery of Zn^2+^ from 4 days to more than 7 days compared to similar hydrogels, so that continues angiogenesis and osteogenesis were able to conduct for a stable period, thus narrowing the recovery time when treating bone defects.

**Fig. 11:**
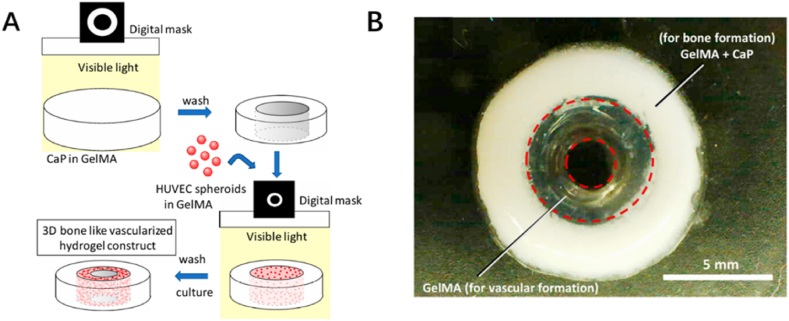
*(A) Schematic illustration of preparation and fabrication for the hydrogel. (B) A photograph for the 3D structure of the hydrogel* [85].

In another study conducted by Anada and coworkers, a fabricated 3D hydrogel that mimicked human bone structure was developed by means of stereolithography (SLA), one of the main bioprinting techniques (Fig. 11B) [85]. The bone-mimetic hydrogel consists of octacalcium phosphate (OCP), spheroids of human umbilical vein endothelial cells (HUVEC), and GelMA hydrogels (Fig. 11A). Detailly, the outer layer of the novel hydrogel is composed of ring-structure GelMA hydrogels embedded with OCP to mimic the cortical shell, and in the center, another GelMA ring containing HUVEC spheroids is substitute for the bone marrow. OCP has the ability to hydrolyze into Ca-deficient HA, which will uptake Ca^2+^ from surrounding environment and release inorganic phosphate ions out. Therefore, the outer GelMA layer involved OCP provided multiply materials in osteogenic reaction and significantly promoted differentiation of stem cells into osteoblasts, which is confirmed from results of the study. HUVECs encapsulated in spheroids greatly enhanced the angiogenic effect of the hydrogel which was reflected from the 3D capillary networks formation. In addition, the condition of capillary sprouting from spheroids was found to be associated with the concentration of GelMA, the lower concentration resulting in more sprouting capillaries, which means the angiogenesis or even osteogenesis can be regulated according to clinical requirements. The novel pattern of two or more different culture niches within a single hydrogel is a breakthrough in designing applied hydrogels, which endows hydrogels multifunctional capacities without the risk of mutual interference between various embedded cells and biofactors.

**Fig. 12:**
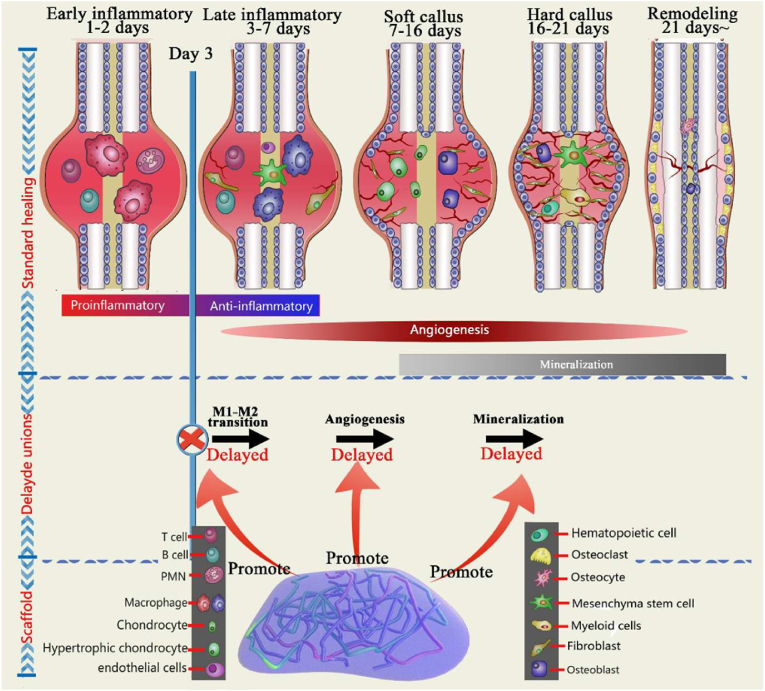
Schematic illustration of the interaction between inflammatory reaction and bone regeneration from the perspective of immunoregulation [90].

Immunoregulation plays a significant role in most of physiological and biochemical reactions in the body including the process of osteogenesis and angiogenesis. Inflammatory cells secrete pro-inflammatory factors in the initial stages of repairment. Only after the reduce of inflammation, which is caused by released anti-inflammatory cytokines, can the repairment of bone tissues is activated [86]. In other words, the progress of promoting bone remodeling highly depends on the control of local inflammation (Fig. 12) [87]. Macrophages, which are mainly classified into classically activated macrophages (M1) and alternatively activated macrophages (M2), are widely involved in the process of regulating inflammation [88]. They will transform into the other phenotype under specific environment to display distinct functions. M1 macrophages can produce pro-inflammatory factors such as IL-12, tumor necrosis factor a (TNF-a), but M2 macrophages inhibit inflammation through secreting TGF-β, IL-10 [89]. Moreover, M2 phenotype macrophages have been reported to promote wound healing and angiogenesis by secreting BMP-2 and VEGF. Therefore, regulating the transition of macrophages into M2 phenotype is a promising approach in bone tissue reconstruction.

Qiu et al. prepared an injectable periosteal extracellular matrix (PEM) hydrogel derived from porcine decellularized periosteum [90]. This fabricated hydrogel demonstrated a remarkable effect on activating M1-M2 shift, because a great quantity of CD206^+^ (M2 marker) cells were observed surrounding the defect while M1 macrophages still remained in the majority at the same site in controlled hydrogels. At the same time, PEM hydrogel managed to form more vessels in the calvarial defect model than controlled groups, and the diameter of vessels increased rapidly during the following days. The angiogenesis effect was consistent with the upregulation of osteogenic genes in the osteoblasts cultured on PEM hydrogel. In another study, Ji and coworkers combined a mesenchymal stem cell (MSC)-encapsulated thermosensitive hydroxypropyl chitin hydrogel (HPCH) with a poly (ε-caprolactone) (PCL)/nano-hydroxyapatite (nHA) scaffold, thus regulating the immune responses to promote bone tissue regeneration through vascularization and osteogenesis [91]. The encapsulated MSCs not only serve as stem cells to promote differentiate, but also as an immunoregulator to activate M1-M2 transition, increase M2 polarization macrophages at the defect. In addition, HPCP was confirmed to promote VEGF expression, and PCL/nHA stimulated macrophages to secrete both angiogenic factors such as VEGF, PDGF-BB and osteogenic factors BMP-2, PGE-2. Macrophages, especially M2 phenotype macrophages, playing the key role in the immune cascade, indicate a hopeful direction for designing novel hydrogels which enhance osteogenesis indirectly rather than simply incorporating hydrogels with osteogenic cytokines.

Exosomes are one type of small extracellular vesicles (sEVs) with a diameter ranging from 30 to 200 nm. They possess membrane structures and participate a lot in intercellular communication. Exosomes contain complex functional molecules such as proteins, mRNAs, miRNAs, protecting them from degradation [92]. Thus, exosomes are outstanding nanocarriers to deliver specific substances applied in cell-free therapies for various tissue regeneration [93]. For example, Xu et al. took advantage of engineered exosomes to deliver Kartogenin to synovial fluid-derived mesenchymal stem cells in order to treat cartilage degeneration of osteoarthritis [94]. Liu and coworkers reported that miR-181b exosomes can promote the polarization of M2 state macrophages and inhibit inflammation through PRKCD/AKT signal pathway to facilitate osteogenesis [95]. Zhang et al. used an injectable hyaluronic acid (HA) hydrogel to encapsulate umbilical MSC-derived exosomes (^uMSC^EXOs) for sustained release with the composition of nanohydroxyapatite/poly-ε-caprolactone (nHP/PCL) scaffolds [96]. ^uMSC^EXOs represented significant effect on promoting proliferation and migration of endothelial progenitor cells (EPCs) *in vitro* experiments, and in the cranial defect models of rats, ^uMSC^EXOs enhanced angiogenesis and osteogenesis obviously due to the long-duration sustained release. The researchers investigated the mechanisms and revealed that miR-21, which is the most abundant miRNAs in ^uMSC^EXOs, suppressed the NOTCH1/DLL4 pathway that is involved in vascular development and differentiation. Similarly, Wu and his colleagues also introduced a thermosensitive hydrogel loaded with sEVs derived from bone mesenchymal stem cells (BMSCs) [97]. The novel thermosensitive hydrogel is composed of chitosan (CS) and β-glycerophosphate (β-GP). It has the capacity of transforming from liquids state into gel state while the temperature rises from room temperature to body temperature, and sEVs can be released subsequently for bone repair. In vitro experiments, both osteogenic mRNAs (OCN, OPN, RUNX2) levels and angiogenic mRNAs levels (*VEGF, bFGF, ANG-1*) were increased in cultured cells exposed to BMSC-sEVs, and the upregulation effects were highly dependent on the dose of sEVs. These results were consistent with that of animal model experiments, in which sEV@CS/β-GP composite hydrogel performed much better than control group and single CS/β-GP hydrogel. With the assistance of cell transfection techniques and luciferase assays, the significant pro-angiogenesis and osteogenic effects were contributed to the targeted regulation of exosomal miR-21 which is derived from sEVs on the expression of SPRY2, a gene that cause negative effects on vascular growth factors such as VEGF and bFGF. Encapsulating stem cell-derived exosomes or sEVs in hydrogels is an indirect method to accelerate bone rebuilding via immunoregulation and vascular regulation. Compared with directly injection or stem cells transplantation, this therapy can increase the survival rate of sEVs and induce fewer immune responses, greatly improve the efficiency of treating large bone defects.

#### 2.2.3 Bifunctional and multifunctional hydrogels

Current treatment for malignant bone tumors mainly relies on surgical excision and relevant chemotherapy or radiotherapy. However, tumor recurrence due to insufficient clearance of tumor cells, side effects caused by excessive dose of chemotherapy drugs, failure of osteogenesis to repair the large bone tumor defects and other disease-related events such as subsequent infections, postoperative complications, are significantly associated with the prognosis and life qualities of patients.

In the past decades, hydrogels have demonstrated enormous potential not only in eliminating tumor cells but also in the enhancement of bone remodeling. This is attributed to their excellent capacity for loading chemotherapeutic agents or cytokines that facilitate osteogenesis. However, most of the investigations on hydrogels were merely concentrated on just one treating direction — an antitumor hydrogel seldom participates in bone reconstruction, and vice versa. In fact, eliminating tumor cells and accelerating bone repair are two interrelated and successive processes involved in a cohesive treatment system without order of priority. Therefore, there is a need for novel multifunctional hydrogels which can integrate different functions into a single unit to enhance the therapeutic efficiency.

**Fig. 13:**
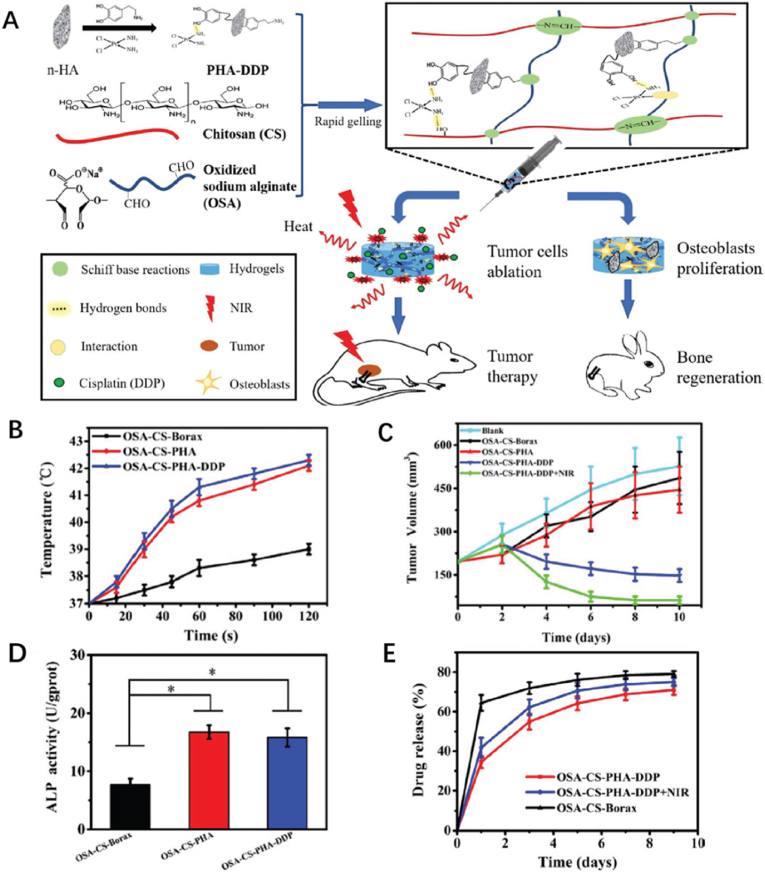
(A) Schematic illustration of the formation of bifunctional OSA-CS-PHA-DDP hydrogels and their medical effects. (B) The photothermal curves showed excellent PTT effect of hydrogels for tumor cells ablation. (C) The tumor growth curves of different hydrogels. (D) The osteogenic differentiation of different hydrogels. (E) The drug release curves of different hydrogels [98].

Recently, Luo and coworkers constructed a bifunctional hydrogel on the basis of oxidized sodium alginate (OSA) and chitosan (CS), with PHA-DDP particles formed by cisplatin (DDP) and n-HA decorated by polydopamine (PDA) via Schiff base reaction (Fig. 13A) [98]. PDA is an excellent photothermal agent that determines the hydrogel to ablate tumor cells under 808 nm NIR laser irradiation, with the synergistic therapeutic effects of loaded DDP (Fig. 13B). There are many functional groups on PDA, which are suitable anchors for DDP. As the temperature increased, the hydrogen bond interactions would be destroyed and DDP were released to induce tumor cells apoptosis (Fig. 13C and E). In terms of the bone repair, n-HA, provided sufficient calcium and phosphorus elements for new bone formation (Fig. 13D). Additionally, oxidized sodium alginate-chitosan-PDA decorated n-HA-cisplatin (OSA-CS-PHA-DDP) hydrogel promoted adhesion and proliferation of cells, which was contributed to PDA for its enhancement effects in corresponding fields [99]. The injectable capacity ensured the hydrogel managed to match irregular bone defects well, and the good biocompatibility and degradability indicated OSA-CS-PHA-DDP hydrogel work well in human body. Similarly, Tan et al. succeeded in developing a hybrid bifunctional hydrogel on the basis of CUR and IR820, which was observed to be efficient for the synergistic treatment of osteosarcoma in 2 days [100]. As the CUR was sustained released simultaneously, a bone reconstruction was promoted.

Although some photothermal agents embedded in hydrogels demonstrate high photothermal transition efficiency, the lack of mechanical strength greatly limits their application in treatment [101]. Chen et al. introduced a bifunctional hydrogel ALG/GelAGE-PDA@DOX via UV and Sr^2+^ double crosslinking [102]. Combining alginate (ALG) and allylated gelatin (GelAGE) hydrogels greatly enhanced the mechanical properties and provided a 3D structure which is similar to ECM environment for loading drugs and cells to promote antitumor and osteogenic effects through sustained delivery. PDA particles that encapsulating DOX served as the major element functioning in synergetic effects between PTT and anticancer drugs. Moreover, this bifunctional hydrogel was proved to promote the proliferation of rBMSCs, and researchers concluded that Sr^2+^ played a significant role in bone regeneration besides participating in crosslink, which was embodied in promotion of osteogenic differentiation and inhibition of osteoclasts activities. On the whole, ALG/GelAGE-PDA@DOX hydrogel exhibited enhanced mechanical properties rather than other hydrogels owing to the double crosslink techniques, with outstanding performance in killing tumor cells and facilitating osteogenesis.

**Fig. 14:**
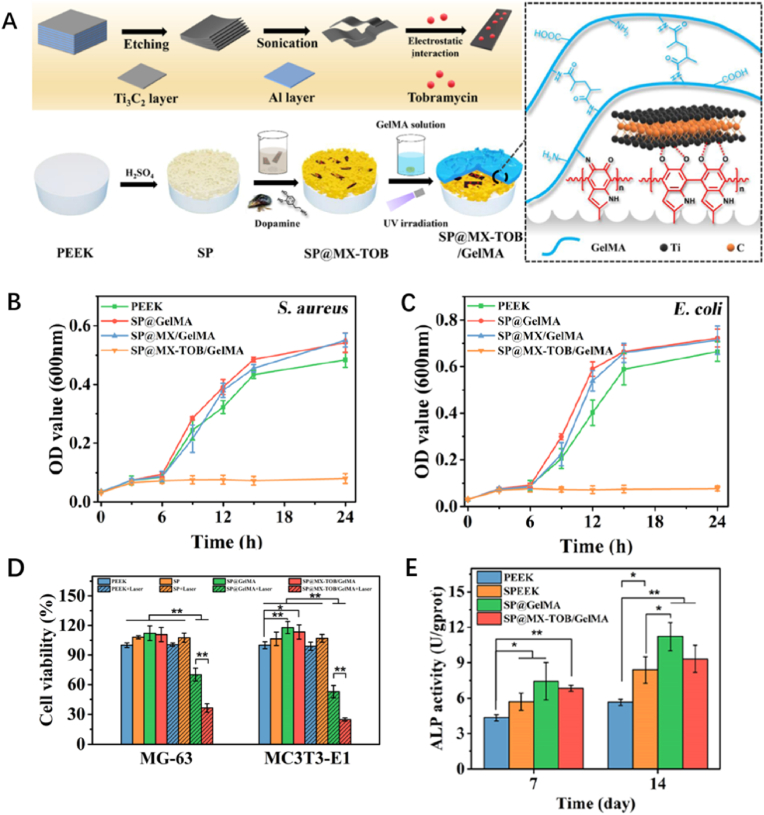
*(A) Schematic image of the formation of multifunctional SP@MX-TOB/GelMA). (B-C) Bacterial growth kinetics curves of S. aureus and E. coli treated with PEEK, SP@GelMA, SP@MX/GelMA, and SP@MX-TOB/GelMA. (D) Cell viability of MG-63 and MC3T3-E1 on different sample groups. (E) ALP expression of cells after 7 and 14 days of culturing* [103].

Postoperative infection is a critical factor influencing the velocity and degree of bone reconstruction [104]. Antibacterial hydrogel has been demonstrated as a satisfactory countermeasure to treat infection and bone defect simultaneously [105,106]. For example, hydrogels incorporated with photothermal agents showed remarkable antibacterial effects and accelerate wound healing when exposed to NIR irradiation [107,108]. Yin et al. synthesized a multifunctional hydrogel based on GelMA hydrogels, consisting of tobramycin (TOB)-laden transition metal carbides and nitrides (MXenes) nanosheets and bioinert sulfonated polyetheretherketone (SP) (Fig. 14A) [103].The novelty of SP@MX-TOB/GelMA hydrogel lies in its unique “dual synergistic effects”. After the coating of PDA, the hydrogel displayed remarkable PTT effects, ablating osteosarcoma cells to the maximal extent due to the synergistic effects of MXenes and PDA (Fig. 14D). In addition, Ti_3_C_2_-based MXenes nanosheets were found to inhibit the growth of bacteria groups through direct contact, and once the loaded TOB releases, it could suppress bacterial protein synthesis via interfering the process of translation, which endow the hydrogel excellent antibacterial effects synergistically (Fig. 14B and C). In vitro experiment, the elevated ALP expression of MC3T3-E1 cultured on SP@MX-TOB/GelMA hydrogel and densely distributed calcium deposition revealed that the active osteogenesis was taking place (Fig. 14E).

Taken together, adding loaded antibiotics on hydrogels to prevent postoperative infections on the premise of ensuring antitumor and osteogenic effects is a promising approach in reinforcing bifunctional or multifunctional hydrogels, which greatly expand the application on malignant bone tumor treatment.

## 3 Discussion and perspective

Therapeutic approaches for malignant bone tumors have experienced significant advancements over the past several decades. This evolution has seen a gradual shift from isolated amputation to the implementation of a multimodal treatment strategy that integrates surgery, chemotherapy, and radiotherapy. [2]. These advancements greatly raised the 5-year survival rate of patients from about 30 % to approximately 70 % [3]. Nevertheless, the study has encountered a bottleneck period recently, leading researchers to invest considerable efforts toward the advancement of implantable biomaterials in the BTE field.

Hydrogel is regarded as one of the most promising injectable materials for encapsulating bioactive molecules and therapeutic drugs owing to its porous network structure and high biocompatibility, which provide a suitable habitat for cells aggregation and propagation [17,35]. In addition, the excellent biodegradation liberates the patients from the pain of a secondary operation. During the degradation process, the encapsulated ions and degraded products will produce a favorable effect on the local lesion and periphery microenvironment, destroying the malignant bone tumor cells or supporting and accelerating the repairment of tissues and wounds [109]. However, as Pan et al. reported, one of the primary challenges that limits the further application of the hydrogel is the relatively lower mechanical property compared with other implant materials [110].

The injectable capacity is a prominent advantage of hydrogel, as minimal invasion can alleviate procedural pain and reduce the risk of subsequent infections. However, nascent injectable hydrogels are limited to loading specific drugs for local delivery without precise control over the release rate and dosage. Thereby it can easily result in a dilemma, insufficient treatment or excessive drug accumulation. The deficiency of antitumor drugs results in the incomplete elimination of tumor cells which may induce relapse. Conversely, superabundant dosages not only hurt surrounding normal tissue cells but also impose an undue burden on the liver and kidneys. Stimuli-responsive hydrogel is a great breakthrough in the study of injectable hydrogels [48]. It enables the sustained drug release tailored to treatment demands through transforming the swelling behavior of hydrogels in response to variations in physical and chemical parameters, particularly temperature and pH values [111].

The vast majority of the raw materials that constitute hydrogels have not shown significant antitumor effect. The responsibility for eliminating tumor cells highly relies on the encapsulated chemotherapy drugs or other supplementary substances such as chemosensitizer and photosensitizer. Chemosensitizer, such as CUR, can significantly improve the efficiency of antitumor agents while relatively reducing drug dosages [112]. This approach minimizes the drug toxicity, thereby alleviating the burden on organs and decreasing potential latent side effects. Photosensitizers play a crucial role throughout the whole process of PTT and PDT. In PTT, photosensitizers convert the energy of light into the form of heat, selectively ablating tumor cells while minimizing damage to surrounding tissues. The incorporation of chemotherapeutic agents can further enhance the synergetic effect, maximizing antitumor efficacy. Apart from the outstanding performance in inhibiting tumor growth and metastasis, hydrogels with PTT effect were also reported to be beneficial to restrain inflammation and decrease the risk of infection through various mechanisms, which may accelerate the healing of bone tissue and wound to avoid delayed union or nonunion. It should be pointed out that PTT may hurt normal cells or tissues if the temperature is solely increased [113], which is determined by the irradiation power and time of NIR [114]. Zhang et al. suggested the temperature of PTT must be in the range of 41 °C–45 °C for clearing tumor cells regardless of healthy cells [115]. Similarly, Liu et al. reported that low-temperature PTT (43–45 °C) may be a viable approach to protect normal cells while killing tumor cells [116]. This method leverages the inhibition of heat shock proteins (HSP), which can diminish the heat resistance of tumor cells. Consequently, this enhancement in sensitivity to thermal treatment improves the efficacy of tumor PTT while reducing the temperature required for effective intervention. Another approach to improve the defects of PTT is to improve the targeting capability of photosensitizers [117]. By conjugating tumor-targeting ligands to photosensitizers, these agents can effectively bind to tumor cells, achieving a higher concentration at the tumor site. Upon exposure to NIR, the temperature at the tumor site rises above that of surrounding healthy tissue, thereby minimizing the risk of inadvertent damage to normal cells. Therefore, selecting appropriate photosensitizer has great significance when assembling hydrogels for PTT. In PDT, photosensitizer participates in the generation of ROS, which directly kills cancer cells and usually prompts the dissociation of hydrogel to release encapsulated drugs, stimulating more ROS generated in turn. Unfortunately, excessive ROS also poses a significant threat to healthy cells and tissues, potentially undermining the efficacy of co-loaded drugs and biological agents. Therefore, an appropriate dispersion of photosensitizers and an ROS responsible modulation system should be taken into consideration when designing PDT hydrogel for treating malignant bone tumors [117].

The process of bone reconstruction is intricate and dynamic, involving a variety of bioactive factors and systems within the human body. Cytokines that directly stimulate bone growth, such as BMP, TGF, IGF-1 are the first choice when assembling hydrogels [80]. Similarly, the pro-angiogenesis cytokines like VEGF and TNF-α are particularly appealing, as the early vessel formation is quite vital for osteogenesis [81]. These cytokines interact synergistically to create a conducive microenvironment for new bone growth, especially in those hydrogels whose composition and structure are mimicking the ECM [85,118,119]. In addition, peptides and bioproteins have emerged as significant research focal points in the fields of osteogenesis and angiogenesis [120,121]. Cheng et al. developed an injectable polypeptide-protein hydrogel that exhibits vascularization and antibacterial properties, which is composed of K_2_(SL)_6_K_2_(KK) polypeptide, thiolated bovine serum albumin protein and silver ions [122]. Hao et al. reported that supramolecular peptide nanofiber hydrogels can effectively emulate the natural bony extracellular matrix in three key aspects: structural features, biochemical functions, and mechanical properties [123]. These characteristics suggest their potential for applications in bone repair and regeneration. In comparison to cytokines, peptides possess an advantage in terms of flexible design and controllable synthesis, allowing for precise alignment with medical requirements. Both cytokines and peptides are highly appealing for encapsulation in hydrogels aimed at promoting osteogenesis and angiogenesis.

The immune system plays a vital role in both the antitumor response and the process of osteogenesis. Immune checkpoint blockade therapies targeting PD-1 and TIM3, along with cellular immunotherapies primarily focused on T cells, are significant advancements in the field of tumor immunotherapy [76,113,124]. CD8^+^T cells and memory T cells are crucial in killing tumor cells. Encapsulating appropriate PD-1/PD-L1 inhibitors or agents that can activate T cells, as well as counteract immunosuppression within TME in a suitable hydrogel represents a promising strategy. Furthermore, macrophages are actively involved in the modulation of TME. M1 macrophages can secrete pro-inflammatory molecules such as IL-1, IL-18, and TNF-α, which exert anti-tumor effects. In contrast, M2 macrophages are associated with promoting carcinogenesis [71]. However, pro-inflammatory M1 macrophages may contribute to chronic inflammation and hinder the resolution of inflammation in the early stages of osteogenesis if an imbalance between M1 and M2 macrophages occurs [86,87,91]. This requires a precise modulation of macrophage states throughout the entire antitumor and osteogenesis processes. A successful hydrogel designed for immunotherapies, which enhances the antitumor effect and facilitates the reconstruction of bone defects caused by malignant bone tumors, is intrinsically linked to the aforementioned elements.

Hydrogels have experienced significant evolution, transitioning from basic, single-function materials to sophisticated multifunctional systems over the decades. Initially, during the mid-20th century, hydrogels primarily fulfilled passive roles such as hydration and biocompatibility. The period spanning the 1970s to the 1990s witnessed the emergence of stimuli-responsive smart hydrogels, which enabled dynamic functions including controlled drug release. Since the 1990s, advancements in nanocomposites, biofunctionalization, and hybrid networks have further enhanced their multifunctionality. Bifunctional and multifunctional biomaterials are the most advanced designs currently, which are superior than traditional ones in achieving various therapeutic objectives within a single scaffold. These biomaterials can exhibit a range of functions, such as antitumor, pro-osteogenesis, pro-angiogenesis and anti-infection properties, thereby maximizing the utility of limited resources [102,125]. The distinctive capability to load agents endows hydrogels with bifunctional qualifications. The antitumor effect is primarily achieved through local hyperthermia induced by photothermal effects combined with synergistic therapies; this approach is particularly advantageous as conventional chemotherapy often necessitates a substantial quantity of drugs that may adversely affect the activity of bioactive ions. The major task in constructing such bifunctional hydrogels is effectively coupling appropriate photosensitizers and osteogenic factors while preserving their biological activity. When designing these hydrogels, it is crucial to prioritize considerations regarding excessive heat, whether from elevated temperatures or prolonged exposure during PTT, as well as potential toxicity arising from intermediate products or the materials added themselves. Additionally, some hydrogels have been engineered to function as containers that encapsulate multiple niches, thereby preventing interactions among various drugs. This design allows for the sequential release of different substances in response to changes in temperature or pH, presenting an alternative strategy for hydrogel formulation. Theoretically, bifunctional hydrogels have better prognosis in patients with malignant bone tumors than single hydrogels. The growth and spread of remain tumor cells largely depend on the level of local angiogenesis activity, which may be relatively high in hydrogel promoting angiogenesis singly for bone reconstruction. This generates a hidden danger for malignant bone tumor recurrence or distal metastasis. Bifunctional hydrogels have superior space-time efficiency in solving this dilemma. The hyperthermia mediated by PTT will destroy the cell vitality of tumor cells within a short duration before the angiogenesis activity take place. Even the tumor cells escaped this treatment round will be killed by subsequent PTT. Bone tissue regeneration is a continuous process lasts for a long duration, which is relatively slow and late compared to the elimination of malignant bone tumors.

As Liao et al. concluded, the desired hydrogel for treating malignant bone tumors is supposed to display a targeted and long-lasting antitumor effect, alleviate the treatment-related suffering, and facilitate the bone defect repairment without the need for additional drugs or therapeutic regimens [126]. These clinical demands indicate that hydrogels will be progressively developed into more comprehensive implanted materials, characterized by excellent injectable and degradable property, favorable physical properties and compressive strength, long-lasting and adjustable sustained release capabilities, as well as reduced biotoxicity and immune inflammation. Therefore, future development of bone tumor-targeted hydrogel may primarily focus on multifunctionality. Multifunctional hydrogel is the closest design to an ideal design, which can realize the therapeutic goal to the maximum. This implies that if a particular hydrogel exhibits sufficiently excellent mechanical and chemical properties to function effectively as a filling material or sustained-release platform for treating malignant bone tumors, it has the potential to be the template for mass production. Such a hydrogel could be utilized in various complex cases merely adjusting the loading drugs.

Indeed, there are numerous constraints on the large-scale clinical application of hydrogels. The process of crosslinking, drying, and rehydration must be meticulously optimized and precisely controlled to maintain the stability of hydrogels. Specialized equipment should be customized to ensure uniform mixing of the reaction system when synthesizing hydrogels, which requires a huge investment in chemical industry. Moreover, achieving precise regulation of drug loading efficiency and release kinetics presents a formidable challenge. The interactions between drug molecules and the hydrogel network significantly influence both loading efficiency and release kinetics, which is difficult to regulate in large-scale production. These challenges suggest that the development of optimal hydrogel materials remains a complex and ongoing endeavor. Nevertheless, the fact that significant advancements in hydrogel research has been witnessed by the scientific community, and further breakthrough are being actively pursued with considerable confidence by researchers in this field.

## 4 Conclusion

This review introduces the recent advances in various hydrogels applied in treating malignant bone tumors, with particular emphasis on the importance of bifunctional hydrogels that exhibit notable antitumor properties and osteogenic capabilities simultaneously. The combination of PTT with chemotherapy agents is considered to be a comprehensive strategy for eradicating tumor cells due to their synergetic effects. Furthermore, the contribution of bioactive mediators in diverse immune responses that enhance antitumor efficacy and facilitate the regeneration of bone defects resulting from tumors should not be overlooked.

## CRediT authorship contribution statement

**Xiangru Qian:** Writing – original draft, Investigation. **Lian Guan:** Writing – original draft, Investigation. **Lei Shen:** Writing – original draft, Investigation. **Chenjun Zhai:** Validation. **Yedong Cheng:** Visualization. **Guoqing Pan:** Writing – review & editing, Supervision, Resources, Conceptualization. **Zhenhuan Jiang:** Writing – review & editing, Supervision, Conceptualization.

## Declaration of competing interest

The authors declare that they have no known competing financial interests or personal relationships that could have appeared to influence the work reported in this paper.

## Data Availability

No data was used for the research described in the article.
